# Tumor stiffness extends its grip on the metastatic microenvironment

**DOI:** 10.1080/23723556.2017.1372866

**Published:** 2017-10-16

**Authors:** Steven E. Reid, Sara Zanivan

**Affiliations:** a Division of Translational Cancer Research, Department of Laboratory Medicine, Lund University, Lund, Skåne, Sweden; b Tumour Microenvironment and Proteomics, Cancer Research UK Beatson Institute, Glasgow, UK; c Tumour Microenvironment and Proteomics, Institute of Cancer Sciences, University of Glasgow, Glasgow, UK

**Keywords:** CYR61/CCN1; blood vessels; stiffness; cancer metastasis; proteomics

## Abstract

The increased stiffness of a tumor triggers a multitude of responses that aid cancer cell dissemination. Stiffness-induced expression of CCN1 mediates autocrine signaling in the endothelium to upregulate N-Cadherin levels. This permits more stable interactions with cancer cells and increases their ability to spread into the circulation.

Metastasis is the leading cause for cancer related deaths. The vascular endothelial cells (ECs) are key players in this process by providing a major route for cancer cells to escape the primary tumor and metastasize to distant sites. Cancer cell intravasation is facilitated by leaky tumor vessels and by the expression of cellular adhesion proteins on the cancer and endothelial cells. However, the effect of the tumor microenvironment on the ECs and the impact this has on metastasis is not well defined.

The extracellular matrix (ECM) is heavily remodeled during tumor development and is dynamic both in its composition and structural properties. Alterations in the ECM during tumor progression often lead to increased tumor stiffness, promoting primary tumor growth and metastasis by directly affecting cancer cells through the activation of integrin signaling.^1^ Increased matrix stiffness also helps initiate and maintain the activated cancer associated fibroblast phenotype, which in turn promotes cancer dissemination.^2^ The oncogene transcriptional coactivator YAP1 is a stiffness sensitive transcription factor, whose classical targets include the protein CYR61 (also known as CCN1), which is a secreted matrix-bound protein upregulated during inflammation, wound healing, tissue repair and tumorigenesis. CCN1 is crucial in physiological angiogenesis and vascular development and also possesses pro-tumorigenic and pro-metastatic roles as a ligand itself or by binding growth factors, metalloproteases (MMPs) and cytokines to modulate their signaling.^3^ CCN1 acts on cancer cells, fibroblasts, ECs and immune cells as a non-RGD-containing ligand of integrins αvβ3, α6β1, αvβ5, αMβ2 and αIIbβ3^3,4^. In ECs the dominant receptor is αvβ3, mediating migration, survival and tubule formation.^5^ The context specific signaling of CCN1 is likely due to its modular domain structure, enabling multiple integrin and co-receptor engagement at once, triggering a unique downstream signaling.^3,4^

## Tumor stiffness: A sticky situation

To assess how tumor matrix stiffness affects ECs, we cultured primary human umbilical vein endothelial cells (HUVECs referred to as ECs) on polyacrylamide gels representing physiological (400 Pa) or tumor stiffness (22000 Pa).^1^ Mass spectrometry-based proteomic analyses revealed approximately 250 of the 5,400 proteins quantified were regulated with stiffness (Datasets are publically available^6^). Several transmembrane cell adhesion proteins were upregulated with stiffness, in particular N-Cadherin. Unlike VE-Cadherin at cell-cell contacts, N-Cadherin is dispersed over the cell surface, mediating contacts with other cell types such as pericytes. In a tumor context, epithelial to mesenchymal transition (EMT) typically yields metastatic cancer cells expressing elevated N-Cadherin, which plays a key role in the process of transendothelial migration (TEM) during intravasation and extravasation.^7^ We hypothesized that the alteration in vascular N-Cadherin may affect the ability of cancer cells to cross the endothelial barrier.

CCN1 was significantly upregulated at high stiffness in cultured ECs and in an orthotopically transplanted breast cancer model. CCN1 was also found to be upstream of stiffness-induced N-Cadherin expression, which is partly dependent on β-catenin activity.

We recapitulated the initial stage of metastasis in vitro and measured the ability of N-cadherin expressing cancer cells to stably adhere to the endothelium and to transmigrate through a microvascular endothelial monolayer. ECs expressing CCN1 at high stiffness increased the binding of cancer cells via N-Cadherin interactions, and CCN1 loss prevented cancer cell binding and TEM. Furthermore, intravital imaging analysis of the interaction between fluorescently labelled B16F10 cancer cells and the vasculature demonstrated that loss of endothelial CCN1 reduces cancer cell attachment to the endothelium, whilst increasing motility along the blood vessels. Additionally, injection of a soluble Cre protein intravenously into *Cyr61^loxP/loxP^* mice induced a partial vascular recombination. Together with the B16F10 subcutaneous syngeneic tumor model, *Cyr61 *knockout resulted in fewer lung metastases and detectable circulating cancer cells, without affecting primary tumor growth, vascularity, hypoxia or pericyte coverage. Conversely, incidence of lung metastases when B16F10 cells were intravenously injected was not affected in an EC-specific *Cyr61 *knockout mouse model.

**Figure 1.**
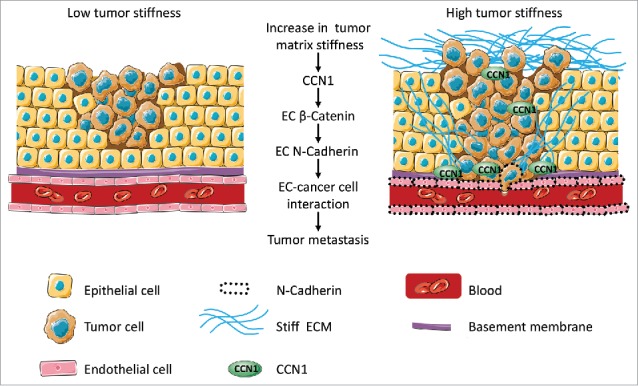
Role of endothelial CCN1 in metastasis. We propose that tumor stiffness induces protein CYR61 (indicated as CCN1) expression, which mediates β-catenin signaling and N-Cadherin expression in the endothelium. N-Cadherin engagement with the cancer cells facilitates their adhesion and transendothelial migration into the bloodstream subsequently increasing the likelihood to generate metastasis. ECM = extracellular matrix. EC = endothelial cell. Images adapted from Servier Medical Art http://smart.servier.com/.

This data enabled us to draw a model through which endothelial CCN1 controls cancer cell metastasis. The desmoplastic reaction that accompanies tumor aggressiveness induces increased levels of endothelial CCN1, which, in turn, upregulates the N-Cadherin available at the surface of the blood vessels. Endothelial N-Cadherin facilitates homophilic heterotypic interactions with aggressive cancer cells that have undergone EMT thus facilitating their intravasation in the blood stream to form distant metastasis (Figure 1).

Other cell-cell adhesion proteins were also increased with matrix stiffness in the ECs, some of which have been implicated in EC-leukocyte interactions. For example CD166 antigen (also known as ALCAM) and poliovirus receptor, which are both crucial in TEM of monocytes.^8,9^ Moreover, high levels of ALCAM in tumor ECs mediate intratumoral infiltration of T-regulatory cells.^10^ One could speculate that increased stiffness could affect immune cell infiltration into the tumor and thus impact tumor progression.

This recent work^6^ was the first to establish that tumor stiffness could influence the endothelium in such a way to aid cancer metastasis. Since CCN1 is extracellular and binds multiple cell specific integrins, it is conceivable that therapeutically targeting CCN1 would also impact other cell types with unknown consequences. Further studies are required to fully understand this interaction and unlock to the potential for therapeutic intervention.
